# Burns

**Published:** 1828-06

**Authors:** 


### BURNS.

*Cases of Burns treated with Cotton, by* Dr. Anderson, *at the* Glasgow Royal Infirmary.

About the year 1823, I admitted several cases of burns into the Glasgow Royal Infirmary, to which cotton had been rudely applied previous to admission. In these cases, the cotton was retained on the parts for trial, but without the expected effect; for in none of them had there been any care taken, either in preparing or in properly applying the remedy; on both of which circumstances I am now convinced that much of the success depends. I have since seen a great many cases of the same kind; and others have come to my knowledge, in which, from the improper application, or the too early removal of the cotton, it has failed entirely in being useful.

Nothing, however, can be more satisfactory than the evidence in favor of this plan, when rightly adopted. The first case, indeed, which determined me to use it was one in which the comparative merits of the old and new treatment were very well seen.

I.—A healthy girl was admitted into the Royal Infirmary, on account of a vesicated burn, occupying both legs, the back of the thighs, and part of the nates. Previous to her admission, the right leg had been dressed with carded cotton wool, which having absorbed the discharge, had now formed a close covering to the whole sore. The left leg had been daily dressed with the Linimentum Aquæ Calcis, and discharged healthy pus. She had little uneasiness from the right leg, and the cotton was not removed until the third week, when a new and sound skin presented itself.

Poultices were first applied to the left leg, which, however, continued inflamed and acutely painful. It was afterwards treated by a great variety of applications, according to its particular state and appearance. At the end of the second month there were still several troublesome ulcers in the seat of the injury, which, although frequently much reduced in size, did not cicatrise until the middle of the third month, when she was at length dismissed. The state of both limbs in this case corresponded so nearly as to afford a pretty correct comparison; and the cotton was so obviously preferable to the usual applications, that I determined to try it more fully.

II.—The next case was much more severe. This was a female, aged twenty, whose clothes had caught fire while she was asleep in a state of intoxication. The face, neck, superior extremities, trunk anteriorly and posteriorly as far as the loins, outside of the hips, inside of the thighs, and about the labia pudendi, were much burnt and sphacelated. The mental faculties were not impaired; but the pulse was imperceptible at the wrist, and the action of the heart was found by the stethoscope to be quick and very feeble. She had rigors, a cold skin, and occasional vomiting. Laudanum, ether, and brandy, were given, and the warm bath seemed in this case to be both agreeable and useful. Still she was in great misery; and it was not until the whole injured surface had been carefully enveloped in cotton that she stated herself to be much relieved. The pulse and heat were gradually restored; the vomiting ceased, and her countenance expressed a corresponding increase of comfort. In this state she continued for two days. There was little local pain, and the parts were not touched; but at the expiration of this time she died, as I fully expected, from the severity of the injury. No inspection of the body was obtained.

Here there was at least no discouragement to the repetition of the same treatment. — dead

III.—Another case, in a girl of ten years of age, corresponded in most particulars with that just noticed. In this case the parts were all covered with a thin layer of what is called patent or Liverpool lint, and over this cotton wool was applied, so as to afford protection against pressure. This patient said that the dressings gave instant relief; and no one who saw her before and after their application could doubt this for a moment. In this, as in the former cases, however, the injury was far too great to allow of recovery. The lint was not removed from any part, and she continued easy, and even comfortable, until the fourth day, when delirium came on, and she died. The brain was somewhat softer than usual, and presented numerous bloody points when cut. There were several ounces of serum effused at its base, and the veins of the pia mater were turgid. No morbid appearance was seen in the thorax or abdomen.

These cases showed the primary effects of the remedy in soothing and allaying pain, and the inexpediency, as well as the inutility, of frequently removing the dressings.

IV.—The next case was that of a collier, from an explosion of fire-damp, by which the face, left arm and forearm, part of the right arm, and both ankles, were scorched and vesicated. The cotton was applied immediately on his admission. He had several times been burnt before, and he now expressed in strong language the relief he experienced; and said that he had never, on former occasions, even when the injury was less, felt nearly so easy as under the present treatment. In a fortnight the face was well, without any mark, and without removing the cotton until it came off of itself. About the tenth day I examined the state of the arms, which I now think was improper. This was not repeated, however, and at the end of the fourth week he was dismissed perfectly cured.

V.—A labourer, twenty-one years of age, was next admitted, five hours after the explosion of gas in a coal-pit, by which the whole of the face, right arm, forearm and hand, left forearm and hand, and middle third of both legs, were severely burnt. The parts were quite covered with vesications, except on the hands, where the thick cuticle was detached, and lay loosely upon the cutis, which was inflamed and moist. He complained of excessive pain in the injured parts. They were all carefully put up in cotton, and no point was left uncovered. On the following day, he said he had slept well, and was much relieved, being free of pain. Suppuration took place in a few days; but, although the discharge through the cotton was very considerable, the first dressings were not removed from the limbs until the thirteenth and fourteenth days, and not until the fifteenth day from the face.

They had formed a complete case for the tender parts, coming from the hands exactly in the shape of a glove, and like a mask from the face, so as to form very good specimens of the operation of the remedy.

The aspect of the parts at the first dressing was worthy of particular attention, in reference to the difference they exhibited from burnt surfaces under any other mode of treatment. Many inches of new skin had already been formed along the whole circumference of the sores. The surface interior to this, which could scarcely be called a sore, and certainly not an ulcer, was, throughout its whole extent, on a level with the adjoining sound skin; and, although inflamed and discharging pus, it could not be said that there was any solution of continuity. At first sight, the parts appeared to be covered with numerous small points of granulation; but, from careful examination, I am inclined to think that these points are not properly granulations, but only the natural papillæ of the skin, denuded of cuticle, inflamed and enlarged, so as to present a granular appearance. In this opinion I am strengthened from observing the subsequent process of cure, which, except in cases of deep injury, always takes place without any appearance of cicatrization.

And here it is that the chief excellence of the present plan consists; for it is unnecessary to do more than allude again to the frequent, or rather the constant, occurrence of large, hard, and contracted cicatrizations, even in what are generally looked upon as favorable cases of cure. I am quite sure that, under the usual treatment, by frequent removal of the dressings, of whatever kind they may be, the parts in such a case as this must have become open, granulating sores, of difficult and tedious cure, which would have left scars destroying the motions of the fingers, disfiguring the face, and probably producing ectropion in one or both eyes. The only change observed in this man's face was somewhat curious: he had previously been marked with small-pox, but, after the removal of the cotton, this deformity was scarcely observable.

The cure of this burn may be said to have been accomplished at the end of the fourth week; but some part of the dressings did not separate so soon, and he was detained in the house for some time, partly on this account and partly as an example to the pupils, of the benefits of the practice adopted.

Some care is necessary both in preparing and in applying the cotton. For this purpose it should be finely carded, and disposed in narrow fleeces, so thin as to be translucent; by which means it can be closely applied in successive layers, and is thus made to fill up and protect the most irregular surfaces. The burnt parts, if vesicated, are to be washed with tepid water, and the fluid evacuated by small punctures. Or if more deeply scorched, they may be bathed with a spirituous or turpentine lotion. The cotton is then applied, layer after layer, until the whole surface is not only covered, but protected at every point, so that pressure and motion may give no uneasiness. On some parts it will adhere without a bandage, especially when there is much discharge, but in general a support of this kind is useful. Where the vesications have been broken and the skin is abraded, or where there is sphacelus, more or less suppuration always ensues, and in such cases the discharge may be so great as soon to soak through the cotton and become offensive, particularly in summer; so that it may be necessary to remove the soiled portions. This, however, should be done as sparingly as possible, taking care to avoid uncovering or disturbing the tender surface.

The utility of cotton in burns suggested the propriety of adopting the same remedy in erysipelatous, blistered, and all superficially ulcerated surfaces, from which we do not wish to keep up a discharge.

I have repeatedly tried it in such cases, and I have found it particularly useful in abrasions and excoriations from pressure, whether arising from tight bandaging or from lying too long in one position. Much time is thus saved in healing; and what is of no small moment, much pain and irritation from repeated removals of the dressings is avoided. He only who has suffered from such a cause can set a proper value on the benefits which are thus conferred.*

* Glasgow Medical Journal.

